# Predictors of caregiver depression and family functioning after perinatal stroke

**DOI:** 10.1186/s12887-015-0397-5

**Published:** 2015-07-15

**Authors:** Taryn B. Bemister, Brian L. Brooks, Richard H. Dyck, Adam Kirton

**Affiliations:** Department of Psychology, University of Calgary, 2500 University Drive NW, Calgary, AB T2N 1 N4 Canada; Calgary Pediatric Stroke Program, Alberta Children’s Hospital, 2888 Shaganappi Trail NW, Calgary, AB T3B 6A8 Canada; Neurosciences, Brain Injury and Rehabilitation Program, Alberta Children’s Hospital, 2888 Shaganappi Trail NW, Calgary, AB T3B 6A8 Canada; Departments of Paediatrics and Clinical Neurosciences, Cumming School of Medicine, University of Calgary, 3330 Hospital Drive NW, Calgary, AB T2N 4 N1 Canada; Alberta Children’s Hospital Research Institute, Heritage Medical Research Building, Room 293, 3330 Hospital Drive NW, Calgary, AB T2N 4 N1 Canada

## Abstract

**Background:**

Perinatal stroke is a leading cause of cerebral palsy and lifelong neurological morbidity. Studies on perinatal stroke outcomes are increasing, although examinations of its broader impact on parents and families have been limited. A recent study found that parents of children with moderate and severe outcomes have increased risk for psychosocial concerns, including depressive symptoms and poor family functioning. Other parents adapt remarkably well, but how this occurs is unknown. The primary aim of this study was to examine predictors of parent and family outcomes, namely caregiver depression and family functioning. The secondary aim was to explore potential mediators and moderators of the relationship between condition severity and parent and family outcomes.

**Methods:**

Parents were recruited from a large, population-based perinatal stroke research cohort, and they completed measures assessing their demographics, social supports, stress levels, marital quality, feelings of guilt and blame, psychological well-being, and family functioning. Bivariate analyses compared these variables. Predictor variables, mediators, and moderators were chosen according to the strength of their relationship with the outcome variables (i.e., caregiver depression and family functioning) and theory. Hierarchical regression, mediator, and moderator analyses were conducted accordingly.

**Results:**

A total of 103 parents participated in this study (76 mothers, 27 fathers; mean age of 39.2 years; mean child age of 7.46 years). Condition severity, anxiety, social support, and blame independently predicted caregiver depression while condition severity, stress levels, and marital quality independently predicted family functioning. Blame regarding the cause of their child’s condition also mediated the relationship between condition severity and caregiver depression.

**Conclusions:**

Adverse parental outcomes can be predicted in perinatal stroke populations. Moreover, anxiety and stress management techniques, marital support, and psychoeducation regarding the unpreventable nature of perinatal stroke may be utilized in the future to enhance family outcomes.

## Background

The perinatal period carries a high risk for stroke, occurring in >1:2500 live births [1] and affecting up to 29,500 American children. The consequences are often severe and last a lifetime, including motor impairments (cerebral palsy), epilepsy, behavioural and mental health problems, and cognitive deficits [2]. Studies on perinatal stroke outcomes are increasing, but examination of its broader impact on parents and families has been limited. A recent study of mothers of children with perinatal stroke revealed that many demonstrate resilience with generally favourable psychological outcomes. However, mothers of children with moderate and severe outcomes carry higher rates of depression symptoms, increased stress levels, decreased quality of life, impaired family functioning, and greater marital distress [3]. Comparison of couple dyads within this study demonstrated that fathers may also incur psychological morbidity.

In addition, an objective and validated tool has recently been developed to measure the psychosocial impact of raising a child with perinatal stroke. The APSP Parental Outcome Measure (POM) assesses a wide variety of outcomes including parental guilt and blame regarding the cause of the child’s condition [4]. Parents of children with perinatal stroke often experience misplaced feelings of guilt and blame that may relate to the inability of medical specialists to offer a specific cause of stroke in most cases [5]. Parents may then erroneously assign causation to occurrences around the time of the stroke. For example, mothers may assume that they did something wrong during pregnancy or assign blame to routine events surrounding labour and delivery. This parental guilt and blame may adversely affect parents’ psychological well-being, potentially for decades, and has been observed in other populations [6]. Importantly, such misplaced feelings may be amenable to change through simple psychoeducation regarding the currently unpreventable nature of perinatal stroke [5].

Despite these recent studies, the specific variables that differentiate parents who adapt well from those who do not are yet to be determined. Potential determinants of the psychological well-being of caregivers have been explored in other pediatric conditions, however, and they include child, parent, and psychosocial variables [7, 8]. The most common child variables that predict caregiver well-being are condition severity, behavioural problems, cognitive deficits, and adaptive functioning [7, 9]. Parent variables appear to be more variable as potential determinants of caregiver depression and mental health. Examples include proxies of socioeconomic status (e.g., income level, education level, and occupational status), ethnicity, age, and gender [8, 10–12]. A vast selection of psychosocial variables has been shown to independently predict caregiver depression, including caregiver stress [13], social support [14, 15], and marital quality [11, 16]. Other psychosocial variables that have been associated with caregiver well-being are anxiety [17], guilt [18], self-esteem [15], self-efficacy [7], and coping strategies [7].

Fewer studies have examined predictors of family functioning, despite its relevance to family-centered care and the child’s health and psychosocial functioning [19]. Research to date has demonstrated that family functioning is associated with child, parent, and psychosocial variables. Family distress and functioning can be affected by the child’s condition severity, cognitive deficits, behavioural problems, and motor abilities [20–22]. Less consistent findings exist regarding the impact of demographic variables on family functioning, such as parent age, gender, income, education, and ethnicity [20, 22]. Other studies focus on psychosocial variables like self-esteem [23], positivity [24], and marital status [25] and highlight their contributions to family adjustment.

These studies largely align with the Double ABCX Model [26], an established caregiver stress model that helps explain why some families adapt better than others. This model suggests that caregiver adaptation (“X”) may depend on the combination of the caregivers’ stressors (“A” e.g., child’s condition severity, behavioural problems, and cognitive deficits), available resources (“B” e.g., social support, good marital quality, and stress management), the meaning attributed to the situation (“C” e.g., guilt and blame regarding the cause of the stroke), and their accumulation over time. Based on this model, available resources and attributed meaning may mediate the effects of the child’s disability on parent and family outcomes.

Additional caregiver models and frameworks may inform potential mechanisms of caregiver and family adaptation to raising a child with perinatal stroke [7, 8, 27]. Although variations exist within these models, psychosocial variables (e.g., social support and stress) have been consistently identified as potential mediators of caregiver well-being [7, 27]. For instance, studies have confirmed the role of stress as a mediating variable between pediatric disabilities and parents’ psychological well-being (i.e., pediatric disabilities affect caregivers’ stress levels, which in return affects caregivers’ well-being) [13, 28]. However, studies on the process and mechanisms of caregiver and family adaptation remain scarce, especially with respect to family functioning. They also have never specifically addressed perinatal stroke families.

In addition to caregiver stress models supporting a role for psychosocial variables as mediators, other research sheds light on potential moderators of caregiver well-being (i.e., variables that influence the magnitude of the relationship between pediatric disabilities and caregiver well-being). For instance, Gallagher and Whiteley [29] found that child behavior problems moderated the relationship between stress and physical health among parents of children with intellectual disabilities. The aforementioned findings on mediators and moderators are consistent with Wu and Zumbo’s [30] distinction between the two types of variables; mediators are typically cognitive, affective, physiological, motivational, or social states, while moderators are typically innate characteristics, background variables, or traits.

The primary aim of this study was to examine predictors of well-being among parents and families affected by perinatal stroke. It was hypothesized that child variables (i.e., demographic variables, condition severity, and presence of impairments), parent variables (i.e., demographic variables), and psychosocial variables (i.e., stress levels, anxiety symptoms, social support, marital quality, guilt, and blame) would significantly predict caregiver well-being (depression) and family functioning. A secondary aim was to examine potential mechanisms of caregiver well-being and family functioning by investigating mediators and moderators. It was hypothesized that psychosocial variables would act as mediators and child and parent factors would act as moderators between condition severity and parent and family outcomes.

## Methods

### Procedure

Participants were recruited through the Alberta Perinatal Stroke Project (APSP)’s population-based research cohort (please refer to Bemister, Brooks, and Kirton’s 2014 study [4] for a detailed description of the methodology). APSP consists of more than 180 children 0–18 years of age with clinico-radiographically confirmed perinatal stroke in southern Alberta (i.e., neonatal arterial ischemic stroke, periventricular venous infarction, and arterial presumed perinatal stroke [31]). Parents from APSP who agreed to be contacted for research purposes were informed about the study via telephone or email. All consenting parents were then emailed a link to the questionnaire battery, as well as a reminder in two weeks’ time if the questionnaires were not yet electronically submitted. All participants were given the option to fill-out paper versions of the questionnaires, and they received a $10 eGift card in recognition of their contribution. Participants were excluded from the analyses if they had less than nine years of formal education (excluding schooling prior to four years of age), were unable to fluently read English, and were not in a married or common-law relationship. Ethics approval to collect these data was obtained from the Conjoint Health Research Ethics Board at the University of Calgary (Ethics ID 24421).

### Measures

A total of eight questionnaires were administered as part of an ongoing research project (see Bemister et al. for details [4]). Six of these measures were included in the present study in order to minimize multicollinearity and theoretical overlap among the variables (excluded questionnaires: the Parent Experience of Childhood Illness [32] and the Kansas Marital Satisfaction Scale [33]). The six measures are described below:

#### HADS

The Hospital Anxiety and Depression Scale (HADS) [32] is one of the most commonly used research measures for depression symptoms (HADS-D) and anxiety symptoms (HADS-A). A review of over 750 studies established its reliability and validity, as well as its two-variable structure [33]. Although HADS-D and HADS-A were initially developed for medical patients, they have since been validated in outpatient and community populations [33]. Moreover, these scales have been widely used among parents of children with chronic conditions [13, 34, 35] including perinatal stroke [3]. Unlike HADS-A, HADS-D was shown to be highly sensitive to differences between mothers of children with perinatal stroke and mothers of children with typical development [3]. As such, HADS-D was chosen to be the primary outcome variable.

#### PedsQL FIM

The Pediatric Quality of Life Inventory Family Impact Module (PedsQL FIM) [36] measures the impact of pediatric chronic health conditions on parents’ quality of life and family functioning, creating a total of three summary scores: Health-Related Quality of Life (HRQL), Family Functioning, and Total. The psychometric properties of the PedsQL FIM and its summary scores have been demonstrated in several studies, including studies with families of children with cerebral palsy and birth defects [36], chronic pain [37], and sickle cell disease [38]. The PedsQL FIM has also been used among parents of children with perinatal stroke, and the Family Functioning score was chosen to be the secondary outcome variable based on these results [3].

#### PSS

The Perceived Stress Scale (PSS) [39] measures the extent to which situations are judged as being stressful, uncontrollable, unpredictable, and overloading. The scale’s reliability and validity was demonstrated in its original validity study with three samples (two college and one community) [39]. Since then, the scale has been regarded as an effective tool for evaluating stress in parents of children with disabilities and it is commonly used with such populations [40].

#### DAS

The Dyadic Adjustment Scale (DAS) [41] is one of the most established questionnaires assessing marital and common-law relationships [42]. The scale’s theoretical basis, validity, and reliability are illustrated in several studies examining its psychometric properties [43–45]. Furthermore, the DAS has been widely used among parents of children with cerebral palsy [46], epilepsy [47], and intellectual disabilities [48].

#### POM

The APSP Parental Outcome Measure (POM) measures the psychosocial impact of raising a child with perinatal stroke. The POM yields an overall score with three subscales – Psychosocial Impact, Guilt, and Blame – that were recently validated among parents of children with perinatal stroke [4]. The POM and the outcome variable of interest, PedsQL FIM Family Functioning, are both family impact measures for pediatric health conditions [4, 36]. As such, these scales have substantial theoretical overlap. The POM, however, has relatively unique subscales of Guilt and Blame. These subscales were therefore included in the analysis as predictors of family functioning and caregiver well-being.

#### Demographics questionnaire

The Demographics Questionnaire was created to obtain background information about the parent (e.g., gender, age, ethnicity, education level, occupational status) and child (e.g., condition severity, presence of behavioural and cognitive impairments) as part of an ongoing research project (available as a supplemental document in Bemister et al., 2014 [3]; scale has not been validated). The scale additionally assesses parents’ perceived levels of social support by asking them to rate how helpful the following people are in caring for their child with perinatal stroke on a four-point Likert scale: spouse/partner, child’s siblings, grandparents, and friends. These ratings were combined to create a total social support score between 0 and 16, which was used in the analyses, along with the demographic variables listed above.

### Statistical analyses

Descriptive statistics were first calculated for the sample’s demographic variables. The predictor variables were then chosen according to statistical and theoretical considerations. More specifically, Pearson *r* correlations, analyses of variance (ANOVAs), and scatterplots were conducted to examine the strength of the relationship between expected predictor variables (based on the parent, child, and psychosocial variables reviewed) and the outcomes of interest (caregiver depression and family functioning). The variables that demonstrated the greatest association with caregiver depression and family functioning were included in the hierarchical regression analyses (*p* < .01), with the child and parent variables in the first block and the psychosocial variables in the second block. Separate analyses were conducted for the caregiver outcomes of depression (HADS-D) and family functioning (PedsQL FIM Family Functioning). The assumptions of regression analyses – normality, linearity, and homoscedasticity – were examined through residual scatterplots, and transformations of data were conducted as needed. Multicollinearity and singularity were assessed through the variance inflation factor (VIF) and tolerance values.

In order to further explore the relationship between condition severity and parent and family outcomes, moderator and mediator analyses were conducted using the child and parent variables as moderators and the psychosocial variables as mediators. Moderator analyses were conducted according to Baron and Kenny’s procedure [49, 50]. In brief, multiple linear regression analyses were used to test the interaction between condition severity and the potential moderators (i.e., child and parent variables) on parent and family outcomes. The interaction terms were calculated by centering and multiplying the predictors; they were then entered in the second block of the regression analyses while condition severity and the child and parent variables were entered in the first block.

The mediator analyses were administered according to Preacher and Hays’ procedure using the bootstrapping method [51, 52]. Bootstrapping is a non-parametric mediation analysis that is advantageous over other approaches, particularly with small sample sizes due to its reduced Type 1 error rates and lack of assumption of normality [52]. Using this method, the indirect effects of condition severity on parent and family outcomes through the proposed mediators (i.e., psychosocial variables) were tested. The data were resampled 1000 times to generate a 95 % confidence interval, and a mediator effect was deemed present if the confidence interval did not contain zero (i.e., there was a 95 % chance that the total indirect effect was not zero). IBM SPSS Statistics for Windows Version 20.0 was used for all analyses.

## Results

### Sample

A total of 110 parents of children with perinatal stroke were recruited from APSP and participated in an ongoing research project. Among them, 103 parents completed measures of marital quality and therefore were included in the present study (76 mothers and 27 fathers; mean age = 39.2 years; mean child age = 7.46 years). According to parents’ self-report, the majority of the sample were of Caucasian decent (89.3 %), caring for a child with motor impairments (78.6 %), and a mild condition (66.0 %; validated by the Pediatric Stroke Outcome Measure [53], *n* = 94, Goodman and Krusk’s gamma correlation (γ) = 0.62, *p* < .001). See Table 1 for a summary of the child and parent demographic variables.

**Table 1:** Parent and child demographic variables

Demographic Variables	Frequency (%)
Parent gender	
Male	27 (26.2 %)
Female	76 (73.8 %)
Child gender	
Male	56 (54.4 %)
Female	47 (45.6 %)
Parent ethnicity	
Caucasian/White	96 (93.2 %)
Other	7 (6.8 %)
Child ethnicity	
Caucasian/White	92 (89.3 %)
Other	11 (10.7 %)
Type of perinatal stroke	
nAIS	37 (35.9 %)
APPIS	25 (24.3 %)
PVI	25 (24.3 %)
Unclassified	16 (15.5 %)
Severity of condition^a^	
Mild	68 (66.0 %)
Moderate	31 (30.1 %)
Severe	4 (3.9 %)
Motor impairments	
Yes	81 (78.6 %)
No	22 (21.4 %)
Cognitive impairments	
Yes	33 (32.0 %)
No	70 (68.0 %)
Behavioural impairments	
Yes	22 (21.4 %)
No	81 (78.6 %)
Seizures	
Yes	22 (21.4 %)
No	81 (78.6 %)
Total gross household income (CAD)	
<$70,000	30 (29.1 %)
$71,000–$110,000	28 (27.2 %)
>$110,000	45 (43.7 %)
Parent education level	
Grade school certificate	4 (3.9 %)
High school certificate	17 (16.5 %)
College certificate	35 (34.0 %)
Bachelor’s degree	30 (29.1 %)
Master’s, doctorate, or professional degree	17 (16.5 %)
Psychological concerns present prior to child’s birth	
Yes	21 (20.4 %)
No	82 (79.6 %)
Demographic Variables	Mean (SD), Range
Parent age (years)	39.26 (1.70), 26–59
Child age (years)	7.46 (5.42), 0.5–18
Years since perinatal stroke diagnosis^b^	5.59 (4.58), 0.5–18
Years since first clinical presentation^b^	6.41 (5.00), 1–18

^a^Rating is based on parents’ self-reported perceptions of the severity of their child’s condition

^b^ *n* = 86

### Bivariate analyses

An examination of the relationships between the demographic variables and outcome variables revealed that the strongest relationships (*p* < .01) existed with the following child variables: severity of condition (for caregiver depression and family functioning), presence of cognitive impairments (for family functioning), and presence of behavioural impairments (for family functioning). No parent variables were strongly associated with the outcome variables at *p*-value < .01 (Table 2).

**Table 2:** Bivariate analyses between outcome variables and demographic variables

	*F* value (*p*-value)	
Demographic Variables	HADS-D	PedsQL FIM Family Functioning
Parent gender	.49 (.49)	.74 (.39)
Male		
Female		
Child gender	.02 (.90)	1.76 (.19)
Male		
Female		
Parent ethnicity	.74 (.39)	.05 (.83)
Caucasian/White		
Other		
Child ethnicity	.007 (.94)	.004 (.95)
Caucasian/White		
Other		
Type of perinatal stroke	2.11 (.13)	2.82 (.07)
nAIS		
APPIS		
PVI		
Unclassified		
Severity of condition^a^	7.89 (.001)*	18.82 (<.001)**
Mild		
Moderate		
Severe		
Motor impairments	.21 (.65)	1.91 (.17)
Yes		
No		
Cognitive impairments	4.48 (.04)	13.35 (<.001)**
Yes		
No		
Behavioural impairments	2.15 (.15)	16.75 (<.001)**
Yes		
No		
Seizures	0.03 (.89)	.78 (.38)
Yes		
No		
Total gross household income (CAD)	0.95 (.39)	3.64 (.03)
<$70,000		
$71,000–$110,000		
>$110,000		
Parent education level	.39 (.82)	1.97 (.11)
Grade school certificate		
High school certificate		
College certificate		
Bachelor’s degree		
Master’s, doctorate, or professional degree		
Psychological concerns present prior to child’s birth	4.35 (.04)	2.71 (.10)
Yes		
No		
	Pearson *r* (*p*-value)	
Demographic Variables	HADS-D	PedsQL FIM Family Functioning
Parent age (years)	.015 (.88)	−.02 (.81)
Child age (years)	−.12 (.25)	−.01 (.89)
Years since perinatal stroke diagnosis^b^	−.10 (.34)	.004 (.97)
Years since first clinical presentation^b^	−.10 (.34)	−.08 (.47)

^a^Rating is based on parents’ self-reported perceptions of the severity of their child’s condition

^b^ *n* = 86

*p-value < .01 (two-tailed), **p-value < .001 (two-tailed)

Summary statistics of the psychosocial variables and their correlation to the outcome variables are presented in Table 3. The results demonstrated strong relationships between all of the psychosocial variables and the outcome variables (*p* < .01) in the expected direction. The PedsQL FIM Total and HRQL scores were excluded from these and subsequent analyses because their correlation with Family Functioning exceeded the cut-off of .80 suggested by Sweet and Grace-Martin for multicollinearity (2012, p. 180), Total: *r* = .92; HRQL: *r* = .86.

**Table 3:** Descriptive statistics and Pearson r correlations between psychosocial measures and outcomes variables

		Outcome Variables	
Psychosocial Variables	Mean (*SD*), Range	HADS-D	PedsQL FIM Family Functioning^a^
HADS-A	6.52 (3.72), 0–16	.70 (<.001)**	−.57 (<.001)**
PSS	21.95 (9.18), 5–44	.72 (<.001)**	−.67 (<.001)**
DAS^a^	111.50 (19.07), 65–147	−.62 (<.001)**	.58 (<.001)**
Social Support^a^	8.73 (3.91), 0–16	−.26 (.009)*	.25 (.01)*
POM Blame	7.38 (4.82), 0–19	.41 (<.001)**	−.39 (<.001)**
POM Guilt	5.79 (4.48),0–16	.36 (<.001)**	−.33 (.001)**

^a^Higher scores indicate better functioning with respect to the construct being assessed

*p-value ≤ .01 (two-tailed), **p-value ≤ .001 (two-tailed)

### Depression

#### Regression

The predictor variables included one child variable (i.e., condition severity) and six psychosocial variables (i.e., anxiety symptoms, stress levels, social support, marital quality, guilt, and blame). Examination of the residual scatterplots revealed that the data was highly skewed. As such, the data was transformed using a log10 + 1 transformation, which yielded a normal distribution. In addition, examination of the Tolerance and VIF values for the predictor variables revealed that multicollinearity was not present.

The regression analysis showed that condition severity significantly predicted caregiver depression scores, explaining 10 % of the variance, *F*(1, 101) = 10.76, *p* = .001 (Table 4). Anxiety symptoms (HADS-A), social support, stress levels (PSS), marital quality (DAS), guilt, and blame accounted for an additional 56 % of the variance in caregiver depression scores, *F*(7, 95) = 25.42, *p* < .001 (*F* change = 25.28, *p* < .001). The total variance accounted for in this model was 65 %. However, only condition severity, anxiety symptoms, stress levels, and blame were independent predictors (Table 4).

**Table 4:** Hierarchical regression analyses predicting depression and family functioning

Criterion Variable	Step	Predictor Variable	β	Adj. *R* ^*2*^	*F*	*R* ^*2*^ Change	*F* Change
HADS-D	1	Condition severity	−.31**	.09	10.76**	.10	10.76**
	2	Condition severity	−.16*	.63	25.42**	.56	25.28**
		HADS-A	.36**				
		Social support	−.03				
		PSS	.33*				
		DAS	−.10				
		POM Guilt	−.03				
		POM Blame	.17*				
Family Functioning	1	Condition severity	.39**	.26	12.80**	.28	12.80**
		Cognitive impairments	−.07				
		Behavioural impairments	−.17				
	2	Condition severity	.30**	.63	20.63**	.39	20.99**
		Cognitive impairments	−.07				
		Behavioural impairments	−.09				
		Social support	.02				
		HADS-A	−.13				
		PSS	−.29*				
		DAS	.22*				
		POM Guilt	−.08				
		POM Blame	−.10				

* *p* ≤ .05, ** *p* ≤ .001

#### Mediation

**Fig. 1:**
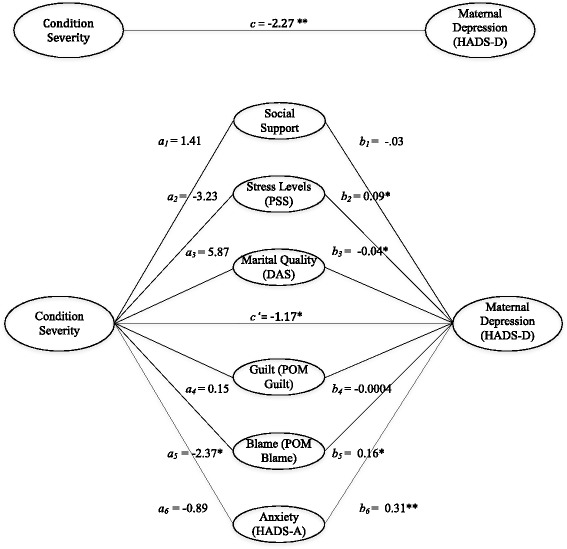
Mediation model for relationship between condition severity, psychosocial variables, and caregiver depression symptoms. *n* = 103. *p ≤ .05. **p ≤ .001

The mediation analysis demonstrated that the psychosocial variables mediated the relationship between condition severity and caregiver depression (total indirect effect = −1.10 with 95 % CI of −2.41 and −0.03; Fig. 1). A closer examination revealed that blame was the only independent variable that significantly mediated the relationship between condition severity and depression (indirect effect = −0.27 with 95 % CI of −0.91 and −0.03).

#### Moderation

No moderators were examined for the relationship between condition severity and caregiver depression, since no child and parent variables were significantly related to HADS-D at *p*-value of < .01 besides condition severity (Table 1).

### Family functioning

#### Regression

The predictor variables included three child variables (i.e., condition severity, presence of cognitive impairments, and presence of behavioural impairments) and six psychosocial variables (i.e., anxiety symptoms, stress levels, social support, marital quality, guilt, and blame). Examination of the residual scatterplots revealed that the data were normally distributed. In addition, multicollinearity was not present as evidenced by the Tolerance and VIF values for the predictor variables.

The regression analysis revealed that condition severity, presence of cognitive impairments, and presence of behavioural impairments significantly predicted family functioning scores, explaining 28 % of the variance, *F*(3, 99) = 12.80, *p* < .001 (Table 4). Social support, anxiety symptoms (HADS-A), stress levels (PSS), marital quality (DAS), guilt, and blame accounted for an additional 39 % of the variance in parents’ family functioning scores, *F*(9, 93) = 20.63, *p* < .001 (*F* change = 17.96, *p* < .001). The total variance accounted for in this model was 67 %, although only three predictors independently reached statistical significance (condition severity, marital quality, and stress level; Table 4).

#### Mediation

The mediation analysis demonstrated that the psychosocial variables did not significantly mediate the relationship between condition severity and family functioning (total indirect effect = 7.04 with 95 % CI of −0.74 and 15.41; figure not shown).

#### Moderation

The moderation analysis found no significant interactions between condition severity and child variables (i.e., presence of cognitive impairments and presence of behavioural impairments) on family functioning (cognitive: *F* change = 0.80, *p* = 37; behavioural: *F* change = 0.16, *p* = .69).

## Discussion

This study is the first of its kind to examine the process of adaptation for parents of children with perinatal stroke. Therefore, it serves to help explain why some parents and families adapt better than others. The primary aim of this project was to examine predictors of caregiver depression and family functioning among parents of children with perinatal stroke. We hypothesized that child, parent, and psychosocial variables would predict caregiver depression and family functioning. Our results provide partial support for this hypothesis. Because no parent variables were strongly associated with caregiver depression or family functioning in the bivariate analyses, they were not incorporated into the regression analysis. Child variables significantly predicted caregiver depression (i.e., condition severity) and family functioning (i.e., condition severity, presence of cognitive deficits, and presence of behavioural problems). All of the examined psychosocial variables were strongly associated with caregiver depression and family functioning; anxiety symptoms, social support, stress levels, marital quality, guilt, and blame predicted caregiver depression and family functioning after controlling for child variables. Moreover, condition severity, anxiety symptoms, stress levels, and blame independently predicted caregiver depression, while condition severity, stress levels, and marital quality independently predicted family functioning.

These results may be interpreted within the context of the Double ABCX Model [26]. Our findings suggest parent and family well-being (“X”) depends on the combination of stressors (“A”; namely the child’s condition severity and presence of behavioral and cognitive impairments), available resources (“B”; social support, good marital quality, and stress management), and the meaning caregivers attribute to the situation (“C”; guilt and blame regarding the cause of the child’s condition), and their accumulation over time. Substantial variation exists in the adaptation of parents and families of children with perinatal stroke, even among children with moderate and severe conditions [3]. Consistent with the Double ABCX Model, parents and families may be buffered from the otherwise negative effects of raising a child with moderate or severe impairments through social support, positive marital relationships, and management of stress levels. These results agree with previous research on pediatric disabilities, demonstrating that satisfaction with social support and network size [7], marital status and marital quality [11, 16, 25], and stress levels and management [13] positively influence caregivers’ mental health. Additional support may be gathered for the Double ABCX model by further evaluating the meaning parents attribute to raising a child with perinatal stroke (e.g., the advantages and disadvantages).

Our exploration of predictors of caregiver depression and family functioning was partially limited by the data collected. Although the child and psychosocial variables we examined accounted for substantial proportions of variance in the models, a host of other variables exist that could impact parent and family well-being. For example, child adaptive functioning and parental positivity or self-esteem were not examined, and may each relate to caregiver well-being and family functioning [15, 54, 55]. These variables are worthy of future investigation among parents of children with perinatal stroke.

In addition, the number of variables included in the regression model was restricted by the sample size and statistical considerations. With the current number of predictor variables, this study has an adequate sample size for multiple regression analyses according to Brace, Kemp, and Sneglar (10:1 ratio of cases to predictor variables) [56]. Nonetheless, this study fails to reach Tabachnick and Fidell’s recommended sample size (N > 104 + m) for testing individual predictors [57]. Thus, some of the predictor variables may have failed to reach statistical significance as predictors of caregiver depression and family functioning due to Type II error. Future research may utilize larger sample sizes to determine this.

The secondary aim of this study was to explore the relationships between condition severity and caregiver depression and family functioning outcomes by examining mediators and moderators. We hypothesized that psychosocial variables would mediate the relationship between condition severity and these outcomes. This hypothesis was not supported for family functioning and partially supported for caregiver depression. The psychosocial variables (anxiety symptoms, social support, stress levels, marital quality, guilt, and blame) were found to mediate the relationship between condition severity and caregiver depression. Blame regarding the cause of the child’s condition was the only independent mediator of this relationship. These results align with the Double ABCX Model [26], as well as other caregiver models and frameworks that emphasize the mediating role of psychosocial variables in parent and family well-being [7, 8, 27]. Our study differs from the existing literature in its emphasis on caregiver blame. Parents may be inclined to blame others or themselves for their child’s condition because the primary cause is usually unknown [5]. Our results provide the first empirical evidence of the aversive effects of caregiver blame on their psychological well-being. Moreover, the average amount of time that has passed since the child’s diagnosis was 5.6 years, implying that some parents carry these feelings of blame for many years. Our clinical experiences support this finding and suggest that such feelings may be altered through careful and deliberate psychoeducation regarding the unpreventable nature of perinatal stroke.

Our study’s moderator effects may have been particularly difficult to detect given the relative homogeneity of the sample [50]. The sample consisted mostly of mothers of Caucasian decent with children who do not have behavioral and cognitive impairments. Thus, underrepresentation of groups, such as fathers, may have minimized the power to detect differences in the bivariate analyses. Underrepresentation of groups in the moderation analyses, such as parents of children with behavioural problems, may have minimized the power to detect significant interactions with the outcome variables. As well, the compounded measurement error of the moderator and predictor variables may have contributed to the null findings. Future studies with greater representations across sociodemographic variables (e.g., gender, age, income, education level, ethnicity, etc.) and condition variables (e.g., presence of impairments) may enhance present knowledge on moderators of parent and family outcomes.

Other limitations of this study are inherent to its study design. For instance, causal inferences cannot be inferred based on the data. Longitudinal research is needed in order to determine the impact of child, parent, and psychosocial variables on caregiver depression and family functioning over time. Such a design would also clarify the direction of the examined relationships. We currently cannot eliminate the possibility that the relationships between the child variables, psychosocial variables, and outcomes operate in the opposite direction than we speculated or in a bidirectional manner. For example, some evidence exists that the relationship between parenting stress and child behavioural problems is bidirectional [58]. Similarly, we must consider the possibility that caregiver blame is caused by depression symptoms when interpreting the results of our study.

Furthermore, our study depended on parents’ subjective reports, which introduces the potential for biased responses and shared variance among the measures. Future studies may benefit from incorporating objective data into the study, such as formal diagnoses of depression or the number of diagnostic criteria met for depressive disorder. Lastly, the generalizability of our results is also limited by the study sample. There is reason to believe that parent and family well-being differs for fathers [59], ethnic minorities [12], and parents with low family incomes [60]. These groups were underrepresented in the current study.

## Conclusions

Despite these limitations, this study has important theoretical and clinical implications. This study contributes to current knowledge about the impact of raising a child with perinatal stroke by identifying mechanisms through which parents and families successfully adapt. Based on our results, medical professionals and support workers who assist affected families should seek to reduce caregiver blame and guilt through simple psychoeducation. Additional priorities for intervention and services may include teaching parents stress-reduction strategies and ways to strengthen their marital relationship when faced with adversity. Consistent with family-centered care models, these results may guide policymaking to provide tailored support for families affected by perinatal stroke. Family-centered care promotes the psychological well-being of both caregivers and affected children [61], and it has been associated with greater satisfaction of services. Thus, our study contributes to the growing literature on the family impact of perinatal stroke by identifying targets for intervention and enhancing knowledge on the caregiver adaptation process.

## Abbreviations

APPIS: Arterial presumed perinatal ischemic stroke
APSP: Alberta Perinatal Stroke Project
AIHS: Alberta Innovates – Health Solution
CI: Confidence interval
CDN: Canadian
CIHR: Canadian Institutes of Health Research
DAS: Dyadic Adjustment Scale
HADS: Hospital Anxiety and Depression Scale -(A = Anxiety, D = Depression)
HRQL NAIS: Health-Related Quality of Life Neonatal arterial ischemic stroke
PedsQL FIM: Pediatric Quality of Life Inventory Family Impact Module
POM: (APSP) Parental Outcome Measure
PSOM: Pediatric Stroke Outcome Measure
PSS: Perceived Stress Scale
PVI: Periventricular venous infarction
SDVIF: Standard deviation Variance inflation factor
VIF: Variance inflation factor
